# Association analysis of KIR/HLA genotype with liver cirrhosis, hepatocellular carcinoma, and NUC freedom in chronic hepatitis B patients

**DOI:** 10.1038/s41598-021-01014-x

**Published:** 2021-11-02

**Authors:** Satoru Joshita, Masao Ota, Hiroyuki Kobayashi, Shun-ichi Wakabayashi, Yuki Yamashita, Ayumi Sugiura, Tomoo Yamazaki, Eiji Tanaka, Takeji Umemura

**Affiliations:** 1 Division of Gastroenterology and Hepatology, Department of Medicine, Shinshu University School of Medicine, 3-1-1 Asahi, Matsumoto, Nagano 390-8621 Japan; 2 Department of Health Promotion Medicine, Shinshu University School of Medicine, 3-1-1 Asahi, Matsumoto, Nagano 390-8621 Japan; 3 Consultation Center for Liver Diseases, Shinshu University Hospital, 3-1-1 Asahi, Matsumoto, Nagano 390-8621 Japan; 4 Department of Life Innovation, Institute for Biomedical Sciences, Shinshu University, 3-1-1 Asahi, Matsumoto, Nagano 390-8621 Japan

**Keywords:** Genetics, Gastroenterology

## Abstract

Natural killer cells are modulated through the binding of killer cell immunoglobulin-like receptors (KIRs) with human leukocyte antigen (HLA) class I ligands. This study investigated the association of KIR/HLA pairs with progression to liver cirrhosis, hepatocellular carcinoma (HCC) development, and nucleot(s)ide (NUC) treatment freedom in hepatitis B virus (HBV) infection. KIR, HLA-Bw, and HLA-C were genotyped in 280 Japanese HBV patients for clinical comparisons. No significant associations of KIR/HLA pairs were detected in terms of liver cirrhosis development. The KIR2DS3 positive rate was significantly higher in patients with HCC (n = 39) than in those without (n = 241) [30.8% vs. 14.9%, odds ratio (OR) 2.53, *P* = 0.015]. The KIR3DL1/HLA-Bw4 pair rate was significantly lower in the NUC freedom group (n = 20) than in the NUC continue group (n = 114) (25.0% vs. 52.6%, OR 0.30, *P* = 0.042). In conclusion, this study indicated remarkable associations of KIR/HLA with HCC development (KIR2DS3) and freedom from NUC therapy (KIR3DL1/HLA-Bw4) in HBV patients, although the number of cases was insufficient for statistical purposes. Additional multi-center analyses of larger groups are needed to clarify whether KIR/HLA pairs play a role in HBV patient status.

## Introduction

Hepatitis B virus (HBV) infection is a global health concern, with possible life-threatening liver infection in both acute and chronic disease forms. There are over 250 million estimated HBV carriers in the world, of whom approximately 600,000 die annually from HBV-related liver disease. Chronic HBV infection often leads to liver cirrhosis and eventual hepatocellular carcinoma (HCC)^1–4^. Accordingly, it is the second-leading cause of liver cirrhosis and cancer in Japan after hepatitis C virus (HCV)^5^. Several factors are reportedly involved in the progression to liver cirrhosis and HCC in HBV patients^6^. In order to prevent disease progression, therapeutic agents including nucleot(s)ide analogues (NUCs) and pegylated interferon are currently available^7–10^. However, since it remains difficult to eliminate the covalently closed circular DNA of HBV in the liver with current standard therapies, a life-long commitment to HBV treatment with NUCs and careful HCC surveillance is required, which may also impose a financial burden and potential drug toxicity^11^. It is of clinical need to identify new factors associated with liver cirrhosis and HCC development towards safe NUC discontinuation.

Natural killer (NK) cells provide rapid responses to viral infection and play a key role in tumor immunosurveillance by directly inducing the death of cancer cells^12^. Accordingly, the functional impairment of NK cells has been associated with chronic disease onset and cancer development. NK cell function is centrally controlled by activating and inhibitory killer immunoglobulin-like receptors (KIRs), which bind to human leukocyte antigen (HLA) class I molecules^13–15^. We earlier demonstrated that KIR/HLA receptor-ligand combinations were associated with chronic liver disease progression and HCC development in HCV-infected patients^16,17^. However, no reports have addressed such associations in Japanese patients with HBV to date even though a few studies suggested such associations in other ethnicities^18,19^.

The present study investigated the association of KIR/HLA pairs with disease progression to liver cirrhosis (Study 1), HCC development (Study 2), and freedom from NUCs (Study 3) in Japanese patients with chronic HBV infection.

## Results

### Patient characteristics

The cohort’s characteristics are summarized in Table 1. Median age was 62 years, and 52.5% of subjects were male. Regarding HBV markers, HBsAg-positive and HBeAg-positive patients totaled 78.9% and 14.3%, respectively. Clinically, 240 patients were at the chronic hepatitis stage and 40 patients were at the liver cirrhosis stage. Of the 40 patients with liver cirrhosis, 12 patients had no NUC treatment history or indication for NUC therapy since they had already achieved an HBsAg loss and HBV DNA level of 0 LIU/mL but were at the liver cirrhosis stage at the time of enrollment. The remaining 28 patients at the liver cirrhosis stage received NUC treatment in 2018. Thirty-nine patients had experienced HCC by the end of 2018. Of the 134 patients (47.9%) with prior or ongoing NUC treatment, 20 patients had achieved freedom from NUCs by the end of 2018.

**Table 1:** Demographic and clinical characteristics of patients.

	Total (n = 280)	Patients with cirrhosis (n = 40)	Patients without cirrhosis (n = 240)	*P*-value	Patients with HCC (n = 39)	Patients without HCC (n = 241)	*P*-value
Age, y	62 (49–71)	72 (64–76)	59 (47–70)	< 0.001	69 (61–77)	59 (48–71)	< 0.001
Male, n (%)	147 (52.5)	30 (75.0)	117 (48.8)	0.002	29 (74.4)	118 (49.0)	0.003
AST, U/L	22 (18–27)	25 (23–30)	21 (17–26)	< 0.001	24 (21–29)	21 (18–26)	0.009
ALT, U/L	18 (14–25)	18 (14–25)	18 (14–25)	0.177	16 (13–23)	18 (14–25)	0.340
PLT, × 10^9^/L	19.3 (15.3–23.7)	13.5 (9.8–17.3)	20.2 (16.5–24.5)	< 0.001	15.0 (11.5–19.0)	20.1 (16.3–24.0)	< 0.001
AFP, ng/mL	2.4 (1.8–4.0)	2.2 (1.7–7.2)	2.5 (1.8–3.7)	0.705	2.1 (1.7–12.3)	2.5 (1.8–3.7)	0.812
DCP, mAU/mL	20.0 (16.0–24.0)	21.0 (15.8–30.5)	19.5 (16.0–23.0)	0.144	21.0 (16.5–32.5)	19.0 (16.0–23.0)	0.148
FIB-4	1.6 (1.0–2.4)	3.2 (2.2–5.3)	1.4 (1.0–2.1)	< 0.001	2.7 (1.8–4.5)	1.5 (1.0–2.2)	< 0.001
APRI	0.29 (0.20–0.40)	0.51 (0.35–0.77)	0.27 (0.20–0.36)	< 0.001	0.40 (0.30–0.68)	0.27 (0.20–0.37)	< 0.001
M2BPGi, -/1 + /2 +	193/71/16	13/15/12	180/56/4	< 0.001	19/13/7	174/58/9	< 0.001
HBsAg positive, n (%)	221 (78.9)	28 (70.0)	193 (80.4)	0.002	33 (84.6)	188 (78.0)	0.348
HBeAg positive, n (%)	40 (14.3)	5 (12.5)	35 (14.6)	0.662	5 (12.8)	35 (14.5)	0.778
HBV DNA, LIU/mL	0.0 (0.0–2.4)	0 (0.0–0.0)	1.3 (0.0–2.5)	< 0.001	0.0 (0.0–1.0)	1.3 (0.0–2.5)	0.001
NUC treatment, n (%)	134 (47.9)	28 (70.0)	106 (44.2)	0.002	31 (79.5)	103 (42.7)	< 0.001
NUC free, n (%)	20 (7.1)	0 (0.0)	20 (8.3)	0.014	0 (0.0)	20 (8.3)	0.014
CH/LC, n	240/40	–	–	–	17/22	223/18	< 0.001
HCC ( +), n (%)	39 (13.9)	22 (55.0)	17 (7.1)	< 0.001	–	–	–

*HCC* hepatocellular carcinoma, *AST* aspartate aminotransferase, *ALT* alanine aminotransferase, *PLT* platelet count, *AFP* α-fetoprotein, *DCP* des-γ-carboxy prothrombin, *FIB-4* fibrosis index-4, *APRI* aspartate aminotransferase to platelet ratio index, *M2BPGi* MAC-2 binding protein glycosylation isomer, *HBV* hepatitis B virus, *NUC* nucleot(s)ide analogue, *CH* chronic hepatitis, *LC* liver cirrhosis.

### Clinical characteristics and KIR/HLA genotyping of patients with HBV-induced cirrhosis (Study 1)

Age, male frequency, FIB-4, APRI, and M2BPGi 1 + or 2 + were significantly higher in patients at the liver cirrhosis stage, while platelet count and HBV DNA were significantly lower (Table 1). To clarify the impact of KIR/HLA pairs on disease progression to liver cirrhosis, KIR and HLA genes were genotyped and their frequencies were compared between patients with and without cirrhosis. No significant differences were detected for any KIR or HLA frequency or KIR/HLA pair frequency between the groups (Table 2).

**Table 2:** Frequency of HLA alleles, KIR genes, and KIR/HLA pairs in patients with and without cirrhosis.

Genetic factor	Patients with cirrhosis (n = 40)		Patients without cirrhosis (n = 240)		OR	*P*-value
	n	%	n	%		
**HLA**						
HLA-Bw4	26	65.0	137	57.1	1.40	0.347
HLA-Bw6	33	82.5	214	89.2	0.57	0.226
HLA-C1	40	100.0	237	98.8	1.19	0.477
HLA-C2	5	12.5	42	17.5	0.67	0.433
**KIR**						
KIR2DL1	40	100.0	240	100.0	0.17	–
KIR2DL2	7	17.5	30	12.5	1.48	0.387
KIR2DL3	40	100.0	240	100.0	0.17	–
KIR2DL4	40	100.0	240	100.0	0.17	–
KIR2DL5	19	47.5	100	41.7	1.27	0.490
KIR2DS1	18	45.0	87	36.3	1.44	0.290
KIR2DS2	8	20.0	33	13.8	1.57	0.301
KIR2DS3	6	15.0	42	17.5	0.83	0.698
KIR2DS4*	22	55.0	98	40.8	1.77	0.094
KIR2DS5	18	45.0	77	32.1	1.73	0.110
KIR3DL1	39	97.5	219	91.3	3.74	0.174
KIR3DL2	40	100.0	239	99.6	0.51	0.683
KIR3DL3	40	100.0	240	100.0	0.17	–
KIR3DS1	19	47.5	95	39.6	1.38	0.345
**KIR/HLA pairs**						
KIR2DL1/HLA-C2	5	12.5	42	17.5	0.67	0.433
KIR2DS1/HLA-C2	2	5.0	12	5.0	1.00	1.000
KIR2DL2/HLA-C1	7	17.5	30	12.5	1.48	0.387
KIR2DS2/HLA-C1	8	20.0	33	13.8	1.57	0.301
KIR2DL3/HLA-C1	40	100.0	237	98.8	1.19	0.477
KIR2DS4*/HLA-C1	22	55.0	96	40.0	1.83	0.075
KIR2DS4*/HLA-C2	2	5.0	21	8.8	0.55	0.424
KIR3DL1/HLA-Bw4	25	62.5	122	50.8	1.61	0.171
KIR3DS1/HLA-Bw4	11	27.5	51	21.3	1.41	0.378

*HLA* human leukocyte antigen, *KIR* killer immunoglobulin-like receptor, *OR* odds ratio.

*Full-length KIR2DS4 was genotyped.

### Clinical characteristics and KIR/HLA genotyping of patients with HBV-induced HCC (Study 2)

Age, male frequency, FIB-4, APRI, and M2BPGi 1 + or 2 + were significantly higher in patients with HCC, whereas platelet count and HBV DNA were significantly lower (Table 1). There were no significant differences in the gene frequencies of HLA-Bw4, HLA-Bw6, HLA-C1, or HLA-C2 in patients with and without HCC. The KIR2DS1 positive rate was significantly higher in the HCC group (53.8% vs. 34.9%; odds ratio [OR] 2.18, *P* = 0.023), the significance of which disappeared in the KIR2DS1/HLA-C2 pair analysis. The KIR2DS3 positive rate was significantly higher in the HCC group (30.8% vs. 14.9%; OR 2.53, *P* = 0.015). No significant differences in KIR/HLA proportions were observed between the groups (Table 3).

**Table 3:** Frequency of HLA alleles, KIR genes, and KIR/HLA pairs in patients with and without HCC.

Genetic factor	Patients with HCC (n = 39)		Patients without HCC (n = 241)		OR	*P*-value
	n	%	n	%		
**HLA**						
HLA-Bw4	25	64.1	138	57.3	1.33	0.422
HLA-Bw6	33	84.6	214	88.8	0.69	0.452
HLA-C1	39	100.0	238	98.8	1.16	0.484
HLA-C2	6	15.4	41	17.0	0.89	0.801
**KIR**						
KIR2DL1	39	100.0	241	100.0	0.16	
KIR2DL2	8	20.5	29	12.0	1.89	0.147
KIR2DL3	39	100.0	241	100.0	0.16	
KIR2DL4	39	100.0	241	100.0	0.16	
KIR2DL5	21	53.8	98	40.7	1.70	0.122
KIR2DS1	21	53.8	84	34.9	2.18	0.023
KIR2DS2	8	20.5	33	13.7	1.63	0.264
KIR2DS3	12	30.8	36	14.9	2.53	0.015
KIR2DS4*	16	41.0	104	43.2	1.09	0.803
KIR2DS5	18	46.2	77	32.0	1.83	0.082
KIR3DL1	35	89.7	223	92.5	0.71	0.548
KIR3DL2	39	100.0	241	100.0	0.16	
KIR3DL3	39	100.0	241	100.0	0.16	
KIR3DS1	21	53.8	93	38.6	1.85	0.072
**KIR/HLA pairs**						
KIR2DL1/HLA-C2	6	15.4	41	17.0	0.89	0.801
KIR2DS1/HLA-C2	2	5.1	11	4.6	1.13	0.877
KIR2DL2/HLA-C1	8	20.5	29	12.0	1.89	0.147
KIR2DS2/HLA-C1	8	20.5	33	13.7	1.63	0.264
KIR2DL3/HLA-C1	39	100.0	238	98.8	0.46	0.484
KIR2DS4*/HLA-C1	16	41.0	102	42.3	0.95	0.879
KIR2DS4*/HLA-C2	2	5.1	21	8.7	0.57	0.449
KIR3DL1/HLA-Bw4	23	59.0	124	51.5	1.34	0.383
KIR3DS1/HLA-Bw4	12	30.8	50	20.7	1.72	0.162

*HLA* human leukocyte antigen, *KIR* killer immunoglobulin-like receptor, *HCC* hepatocellular carcinoma, *OR* odds ratio.

*Full-length KIR2DS4 was genotyped.

### Independent risk factors of HCC development by logistic regression analysis

We conducted logistic regression analysis to identify the independent risk factors of HCC development. Regression analysis model 1 including all indices confirmed that liver cirrhosis stage (hazard ratio [HR] 13.42, *P* < 0.001) and KIR2DS3 positivity (HR: 4.15, *P* = 0.003) were independent risk factors of HCC development (Table 4). Model 2 analysis, which included age ≥ 65 years, male gender, and KIR2DS3, demonstrated age ≥ 65 years (HR 2.87, *P* = 0.006), male gender (HR 2.93, *P* = 0.009), and KIR2DS3 positivity (HR 2.90, *P* = 0.011) as independent risk factors (Table 4).

**Table 4:** Independent risk factors of HCC development in HBV patients by logistic regression analysis.

Risk factor (reference)	Univariate		Multivariate			
			Model 1		Model 2	
	HR (95% CI)	*P*-value	HR (95% CI)	*P*-value	HR (95% CI)	*P*-value
Age ≥ 65 years (< 65 years)	2.88 (1.38–5.97)	0.005	1.93 (0.83–4.51)	0.129	2.87 (1.35–6.12)	0.006
Male (female)	3.25 (1.47–7.16)	0.003	2.04 (0.85–4.92)	0.112	2.93 (1.31–6.57)	0.009
Liver cirrhosis stage (non-cirrhosis stage)	14.89 (6.69–33.14)	< 0.001	13.42 (5.51–32.68)	< 0.001		
HBV DNA positive (negative)	0.34 (0.16–0.74)	0.006				
M2BPGi positive (negative)	2.87 (1.34–6.14)	0.007				
KIR2DS3 positive (negative)	2.73 (1.26–5.92)	0.011	4.15 (1.61–10.74)	0.003	2.90 (1.28–6.58)	0.011

Model 1 included all risk factors in calculations.

Model 2 included age, gender, and KIR2DS3 in calculations.

*HCC* hepatocellular carcinoma *HBV* hepatitis B virus, *KIR* killer immunoglobulin-like receptor, *HR* hazard ratio, *CI* confidence interval.

### Clinical characteristics and KIR/HLA genotyping of patients receiving NUC treatment (Study 3)

Twenty of the 134 patients having been treated with NUCs achieved freedom from NUC therapy by the end of 2018. In terms of background characteristics, there were no significant differences between NUC free and NUC continue patients apart from lower frequencies of HBsAg and HBeAg positivity, no progression to cirrhosis, and no development of HCC in the NUC free group (Table 5). The HLA-Bw6 positive rate was significantly lower in the NUC free group than in the NUC continue group (60.0% vs. 89.5%, OR 0.18, *P* = 0.001). The KIR3DL1/HLA-Bw4 pair was also significantly less frequent in the NUC free group (25.0% vs. 52.6%, OR 0.30, *P* = 0.042) (Table 6).

**Table 5:** Demographic and clinical characteristics of patients receiving NUC treatment.

	Total (n = 134)	Patients achieving NUC freedom (n = 20)	Patients requiring NUC continuation (n = 114)	*P*-value
Age, y	62 (50–70)	65 (54–71)	61 (50–70)	0.545
Male, n (%)	77 (57.5)	10 (50.0)	67 (59.3)	0.464
AST, U/L	22 (18–27)	19 (16–25)	23 (18–27)	0.087
ALT, U/L	17 (13–25)	17 (12–21)	18 (13–25)	0.399
PLT, × 10^9^/L	18.7 (14.4–22.4)	19.4 (14.8–22.1)	18.6 (14.4–22.7)	0.903
AFP, ng/mL	2.4 (1.8–3.7)	2.7 (1.8–3.4)	2.3 (1.8–4.0)	0.622
DCP, mAU/mL	20.0 (16.5–24.5)	19.0 (15.8–21.0)	20.0 (17.0–25.0)	0.391
FIB-4	1.7 (1.1–2.7)	1.6 (1.1–2.4)	1.7 (1.1–2.7)	0.851
APRI	0.30 (0.20–0.43)	0.26 (0.18–0.40)	0.31 (0.21–0.43)	0.321
M2BPGi, -/1 + /2 +	87/38/9	13/6/1	74/32/8	0.939
HBsAg positive, n (%)	117 (87.3)	11 (55.0)	106 (93.0)	< 0.001
HBeAg positive, n (%)	109 (81.3)	0 (0.0)	25 (21.9)	0.025
HBV DNA, LIU/mL	0.0 (0.0–1.3)	0 (0.0–1.9)	0.0 (0.0–1.3)	0.158
CH/LC, n	107/27	20/0	86/28	0.014
HCC ( +), n (%)	31 (23.1)	0 (0.0)	31 (27.2)	0.004

*NUC* nucleot(s)ide analogue, *AST* aspartate aminotransferase, *ALT* alanine aminotransferase, *PLT* platelet count, *AFP* α-fetoprotein, *DCP* des-γ-carboxy prothrombin, *FIB-4* fibrosis index-4, *APRI* aspartate aminotransferase to platelet ratio index, *M2BPGi* MAC-2 binding protein glycosylation isomer, *HBV* hepatitis B virus, *CH* chronic hepatitis, *LC* liver cirrhosis, *HCC* hepatocellular carcinoma.

**Table 6:** Frequency of HLA alleles, KIR genes, and KIR/HLA pairs in patients receiving NUC treatment.

Genetic factor	Patients achieving NUC freedom (n = 20)		Patients requiring NUC continuation (n = 114)		OR	*P*-value
	n	%	n	%		
**HLA**						
HLA-Bw4	9	45.0	65	57.0	0.62	0.319
HLA-Bw6	12	60.0	102	89.5	0.18	0.001
HLA-C1	19	95.0	113	99.1	0.17	0.687
HLA-C2	6	30.0	17	14.9	2.45	0.099
**KIR**						
KIR2DL1	20	100.0	114	100.0		
KIR2DL2	1	5.0	19	16.7	0.26	0.312
KIR2DL3	20	100.0	114	100.0		
KIR2DL4	20	100.0	114	100.0		
KIR2DL5	7	35.0	49	43.0	0.71	0.504
KIR2DS1	5	25.0	46	40.4	0.49	0.192
KIR2DS2	3	15.0	21	18.4	0.78	0.959
KIR2DS3	2	10.0	23	20.2	0.44	0.444
KIR2DS4*	8	40.0	50	43.9	0.84	0.724
KIR2DS5	7	35.0	36	31.6	1.17	0.762
KIR3DL1	16	80.0	106	93.0	0.30	0.061
KIR3DL2	20	100.0	114	100.0		
KIR3DL3	20	100.0	114	100.0		
KIR3DS1	7	35.0	48	42.1	0.74	0.551
**KIR/HLA pairs**						
KIR2DL1/HLA-C2	6	30.0	17	14.9	2.45	0.099
KIR2DS1/HLA-C2	0	0.0	5	4.4	0.00	0.753
KIR2DL2/HLA-C1	1	5.0	19	16.7	0.26	0.312
KIR2DS2/HLA-C1	3	15.0	21	18.4	0.78	0.959
KIR2DL3/HLA-C1	18	90.0	114	100.0	0.00	0.959
KIR2DS4*/HLA-C1	7	35.0	50	43.9	0.68	0.441
KIR2DS4*/HLA-C2	3	15.0	10	8.8	1.82	0.393
KIR3DL1/HLA-Bw4	5	25.0	60	52.6	0.30	0.042
KIR3DS1/HLA-Bw4	5	25.0	30	26.3	0.93	0.902

*HLA* human leukocyte antigen, *KIR* killer immunoglobulin-like receptor, *NUC* nucleot(s)ide analogue, *OR* odds ratio.

*Full-length KIR2DS4 was genotyped.

### Independent risk factors of NUC treatment freedom by logistic regression analysis

Logistic regression analysis was performed to identify the independent risk factors of NUC treatment freedom. Regression analysis model 1 considering all indices confirmed HBsAg positivity (HR 0.07, *P* < 0.001) and KIR3DL1/HLA-Bw4 positivity (HR 0.25, *P* = 0.040) as negative independent risk factors of NUC treatment freedom (Table 7). Model 2 analysis, which included age ≥ 65 years, male gender, and KIR3DL1/HLA-Bw4 positivity, demonstrated that KIR3DL1/HLA-Bw4 positivity (HR 0.29, *P* = 0.026) was a negative independent risk factor of NUC treatment freedom (Table 7).

**Table 7:** Independent risk factors of NUC treatment freedom in HBV patients by logistic regression analysis.

Risk factor (reference)	Univariate		Multivariate			
			Model 1		Model 2	
	HR (95% CI)	*P*-value	HR (95% CI)	*P*-value	HR (95% CI)	*P*-value
Age ≥ 65 years (< 65 years)	0.97 (0.37–2.52)	0.950				
Male (female)	0.56 (0.22–1.46)	0.234				
Liver cirrhosis stage (non-cirrhosis stage)	NC	0.012				
HBsAg positive (negative)	0.08 (0.02–0.25)	< 0.001	0.07 (0.02–0.25)	< 0.001		
HBeAg positive (negative)	NC	0.027				
KIR3DL1/HLA-Bw4 positive (negative)	0.29 (0.10–0.87)	0.026	0.25 (0.07–0.94)	0.040	0.29 (0.10–0.87)	0.026

Model 1 included all risk factors in calculations.

Model 2 included age, gender, and KIR3DL1/HLA-Bw4 in calculations.

*NUCs* nucleot(s)ide analogs, *HBV* hepatitis B virus, *KIR* killer immunoglobulin-like receptor, *HR* hazard ratio, *CI* confidence interval, *NC* not calculated.

## Discussion

This single-center, cross-sectional study retrospectively examined whether specific KIR/HLA pairs were associated with progression to liver cirrhosis, HCC development, and freedom from NUC treatment in chronic HBV patients. Our results showed that: (1) there was no detectable association of KIR-HLA genotype with liver cirrhosis development, (2) patients with KIR2DS3 had a significantly higher risk of HCC, with no remarkable involvement of any KIR/HLA pair, and (3) patients with the KIR3DL1/HLA-Bw4 pair were significantly more likely to achieve freedom from NUCs. These genotype differences might have affected the clinical course of HBV by stimulating or escaping immune surveillance and other intrahepatic inflammatory processes.

Many factors are involved in the progression to liver cirrhosis in HBV patients, such as the viral factors of HBV DNA, HBsAg level, and HBV genotype, the host factors of gender and age, and such environmental factors as alcohol intake^6^. Among those, age is a strong predictor of significant fibrosis progression^6^; indeed, the patients with cirrhosis in the present study were significantly older than those without. Regarding host genetic factors, HLA class II has been widely analyzed and linked to disease progression^20^. To date, no association studies have considered KIR-HLA combinations in the Japanese. We therefore conducted this investigation to identify associations of such combinations with disease progression to liver cirrhosis. However, no significant relationship was detected among the KIR-HLA combinations tested. In a Gambian population, KIR and HLA class I combinations were also not related to liver cirrhosis^19^, suggesting that the viral, host, and environmental factors mentioned above might have a stronger influence on liver cirrhosis development in HBV. Longitudinal studies with more subjects are needed to clarify these findings.

Regarding host genetic factors for HCC development in HBV patients, a number of candidate genes specifically for HLA class II regions have been investigated by genetic association studies to evaluate their role in HCC susceptibility^20,21^. KIR and HLA class I combination was reportedly associated with HCC development in Chinese while its combination was not related to HCC development in Gambians^18,19^. A genome-wide association study very recently proposed HLA-A*33:03 as a susceptibility allele for HCC in HBV^22^. The present investigation on the impact of KIRs and HLA-Bw or HLA-C on HCC development detected no remarkable KIR/HLA pairs, although a significant association of KIR2DS3 with HCC onset was found. The ligand to KIR2DS3 has not been identified to date. However, KIR2DS3 has been related to colorectal cancer^23^, spontaneous HCV clearance failure^24^, and fatal outcome of Ebola virus infection^25^. Moreover, KIR2DS3 was seen to be expressed at low level on the NK cell surface with unknown function^26^, and might therefore represent a genetic marker for a closely related linked gene with biological effects. Further research is required to confirm this hypothesis.

We found no association among KIR-HLA genotypes with liver cirrhosis development, although a significant association of KIR2DS3 with HCC development was detected. In general, HCC occurs after patients progress to a liver cirrhosis disease status as evidenced in the natural course of HCV^27^, raising the hypothesis that the same KIR/HLA pairs could be risk factors for liver cirrhosis as well as HCC development. However, in addition to liver cirrhosis stage patients, noncirrhotic patients with HBV infection who have such risk factors as a family history of HCC, Asian male over 40 years of age, and Asian female over 50 years of age, can progress to HCC. Indeed, 17 HCC patients at a noncirrhotic stage were included in this study (Table 1), which suggested a risk difference among KIR/HLA genotypes between liver cirrhosis patients and noncirrhotic patients. A larger number of patients is needed for a sufficient sub-group analysis to verify this hypothesis.

Lastly, HBV patients who receive NUCs are expected to continue treatment indefinitely since there is currently no way to completely eliminate the virus from the liver, with discontinuation sometimes leading to virological relapse and hepatic flares. However, some patients are able to control infection without ongoing treatment. We observed that the KIR3DL1/HLA-Bw4 pair frequency was significantly higher in the NUC continue group despite the KIR3DL1 and HLA-Bw4 expression rates being comparable between the groups. This KIR/HLA pair may be a new biomarker for predicting drug continuation or freedom in HBV patients under NUC treatment. Other ligands to KIR3DL1 include HLA-A23, HLA-A24, and HLA-A32 on the HLA-A locus, which have very recently been reported to exhibit a similar biological function to HLA-Bw4, and have thus been termed HLA-A^Bw4^^28^. Associations of the HLA-A genotype with HBV should be addressed.

Based on the above findings, it is possible that particular KIR/HLA pairs play an important role in regulating the innate immune response and tumor surveillance as well as controlling disease susceptibility in HBV-infected patients. The identified KIR/HLA pair presented in this study may represent an effective predictive marker of HCC development after HBV infection, with additional use as a predictor of the efficacy of anti-HBV treatment with NUCs.

There are several limitations to this study. First, as the number of patients with liver cirrhosis, HCC, and NUC freedom were too small for a definitive conclusion, a larger validation analysis with more subjects is needed to confirm our results. Second, this study was cross-sectional and retrospectively designed, and so the criteria for NUC discontinuation were not aligned; patients who achieved NUC discontinuation were defined as belonging to the NUC treatment freedom group as a result of their clinical course. In order to minimize selection bias, a larger number of prospectively enrolled patients is needed to confirm our results regarding an association between NUC discontinuation and the KIR3DL1/HLA-Bw4 pair. Third, this investigation analyzed HLA-Bw4/Bw6 and HLA-C1/C2, but not other HLA class I genotypes. Additional classical HLA class I genotypes, such as HLA-A, and non-classical HLA class I genotypes should be addressed in future studies.

In conclusion, the present study indicated associations of KIR2DS3 with HCC development and the KIR3DL1/HLA-Bw4 pair with freedom from NUCs in HBV patients, although a larger number of cases are required for statistical purposes. Future multi-center analyses are needed to confirm that KIR/HLA pairs play a role in HBV patient status.

## Materials and methods

### Patients

**Figure 1:**
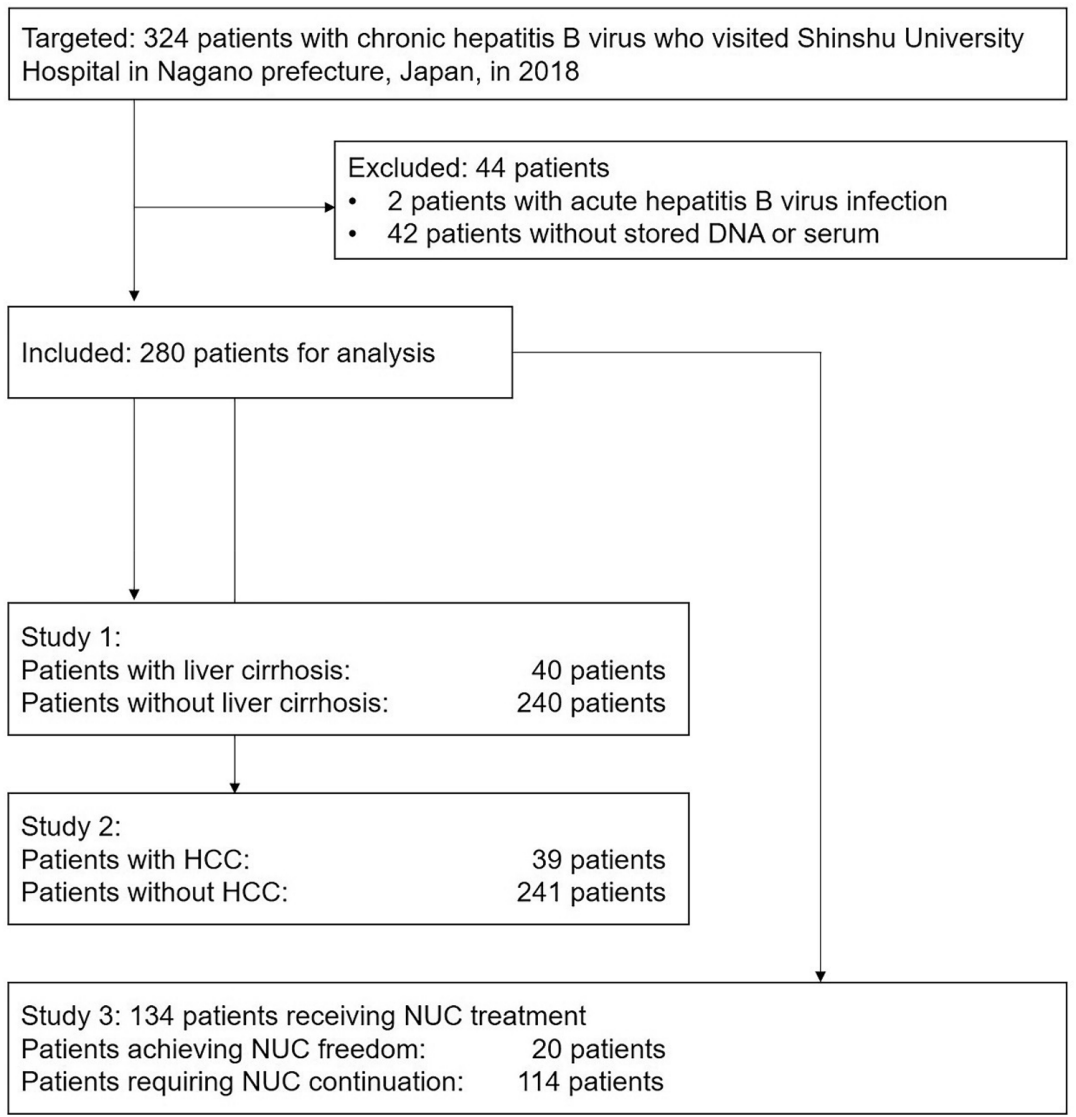
Study participant selection flowchart. HCC, hepatocellular carcinoma; NUC, nucleot(s)ide analogue.

A total of 324 patients of at least 20 years of age who regularly visited Shinshu University Hospital in Matsumoto, Japan, for HBV infection management in the year 2018 (January 1, 2018, to December 31, 2018) were retrospectively targeted. Patients exhibiting other causes of chronic liver disease, such as alcoholic liver disease, non-alcoholic fatty liver disease, primary biliary cholangitis, or autoimmune hepatitis, were not considered. The study participant selection flowchart is depicted in Fig. 1. After the exclusion of 44 cases (2 with acute hepatitis B virus infection and 42 without stored DNA or serum), 280 patients with chronic HBV infection were enrolled in the analysis. The racial background of all patients was uniformly Japanese.

Total bilirubin, alanine aminotransferase, and other relevant biochemical tests were performed using standard methods. HBsAg, HBeAb, and MAC-2 binding protein glycosylation isomer (M2BPGi; Sysmex Co., Kobe, Japan) were measured with a HISCL-5000 (Sysmex Co., Kobe, Japan) system using stocked serum. M2BPGi readings of < 1.00 COI, ≥ 1.00 COI and < 3.00 COI, and ≥ 3.00 COI were judged as negative, 1 + , and 2 + , respectively.

### Determination of liver cirrhosis

Patients with liver cirrhosis were judged as those with histologically proven liver cirrhosis and/or the characteristic clinical signs of advanced liver disease in imaging studies in the year 2018. The number of patients who were diagnosed as being in the liver cirrhosis stage primarily by liver biopsy, ultrasonography, computed tomography, and magnetic resonance imaging were 9, 2, 21, and 8, respectively.

### Determination of HCC

HCC patients were defined as having HCC in the prior quarter century (i.e., between 1993 and 2018) or complicating HCC in 2018. HCC was diagnosed by imaging characteristics, arterial hypervascularity, and venous or delayed phase washout by contrast-enhanced dynamic computed tomography and/or magnetic resonance imaging when a nodular lesion was detected by ultrasonography or a tumor marker was elevated.

### Definition of NUC freedom

Among the 134 patients with prior or ongoing NUC treatment, 20 patients had successfully discontinued therapy by the end of 2018 and were defined as NUC free.

### HLA class I and KIR genotyping

Whole-genomic DNA was extracted from whole blood samples from all participants using QuickGene-800 assays (Fujifilm, Tokyo, Japan). HLA-Bw4, HLA-C1, and HLA-C2 genotyping^29^ as well as KIR genotyping^30^ were performed using the polymerase chain reaction with sequence-specific primers. Regarding KIR2DS4, full-length KIR2DS4 was typed by the polymerase chain reaction with sequence-specific primers in consideration of its functional difference^31^. HLA and KIR typing were used to stratify patients into groups according to predicted KIR-ligand interactions and binding affinities. The KIR/HLA pairs of interest were KIR2DL1/2DS1/2DS4-HLA-C2, 2DL2/3/2DS2-HLA-C1, and 3DL1/3DS1-HLA-Bw4. All genotyping was blinded to clinical variables.

### Statistical analysis

Statistical analysis and data visualization were carried out using StatFlex ver. 7.0.11 software (Artech Co., Ltd., Osaka, Japan). Continuous variables were compared using the Mann–Whitney U test. Categorical variables were evaluated by Pearson’s chi-squared test, Fisher’s exact test, or Yates' continuity correction, as appropriate. Multivariate analysis was performed by means of regression analysis with a stepwise method after categorizing continuous variables to minimize interference. A *P*-value of < 0.05 was considered statistically significant.

### Ethical statement

This investigation was conducted according to the guidelines of the Declaration of Helsinki and approved by the Ethics Committee of Shinshu University School of Medicine (protocol code 302 approved on October 10, 2010, and 527 approved on August 10, 2015).

### Informed consent

Informed consent was obtained from all participants involved in this study.

## Abbreviations

HBV: Hepatitis B virus
HCC: Hepatocellular carcinoma
HCV: Hepatitis C virus
NUC: Nucleot(s)ide analogue
cccDNA: Covalently closed circular DNA
NK: Natural killer
KIR: Killer immunoglobulin-like receptor
HLA: Human leukocyte antigen
M2BPGi: MAC-2 binding protein glycosylation isomer
OR: Odds ratio
HR: Hazard ratio
